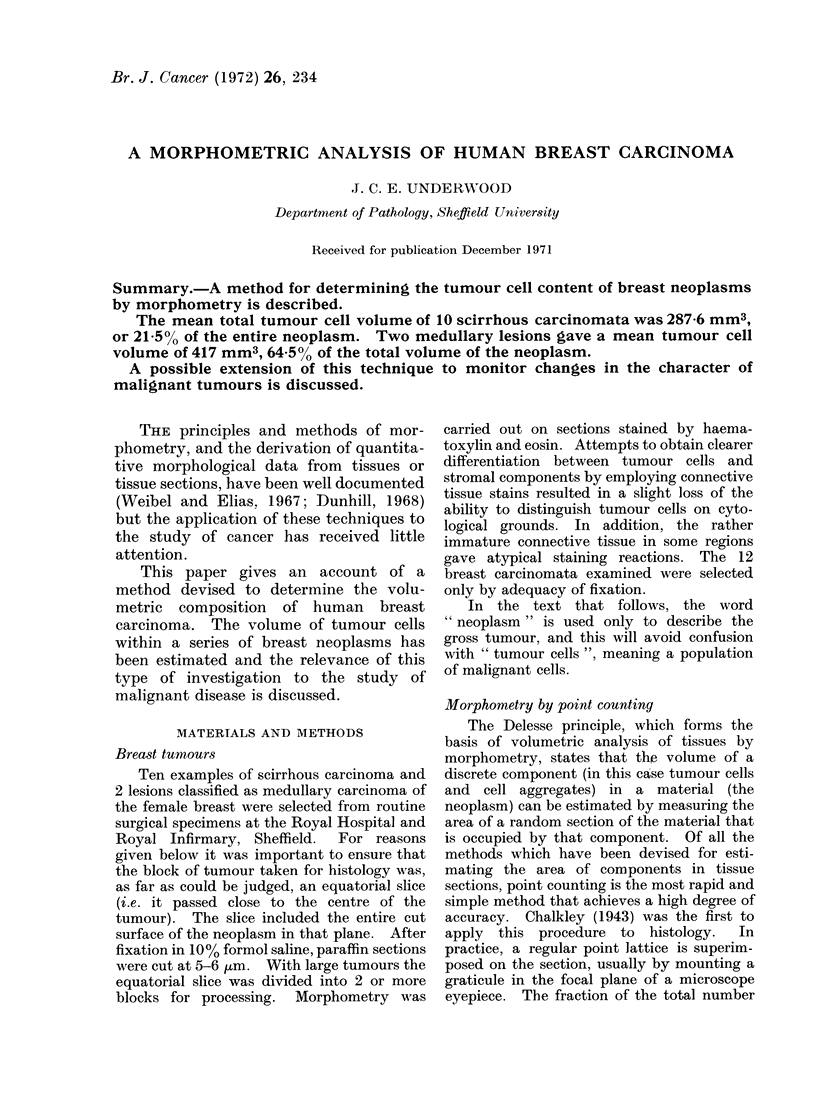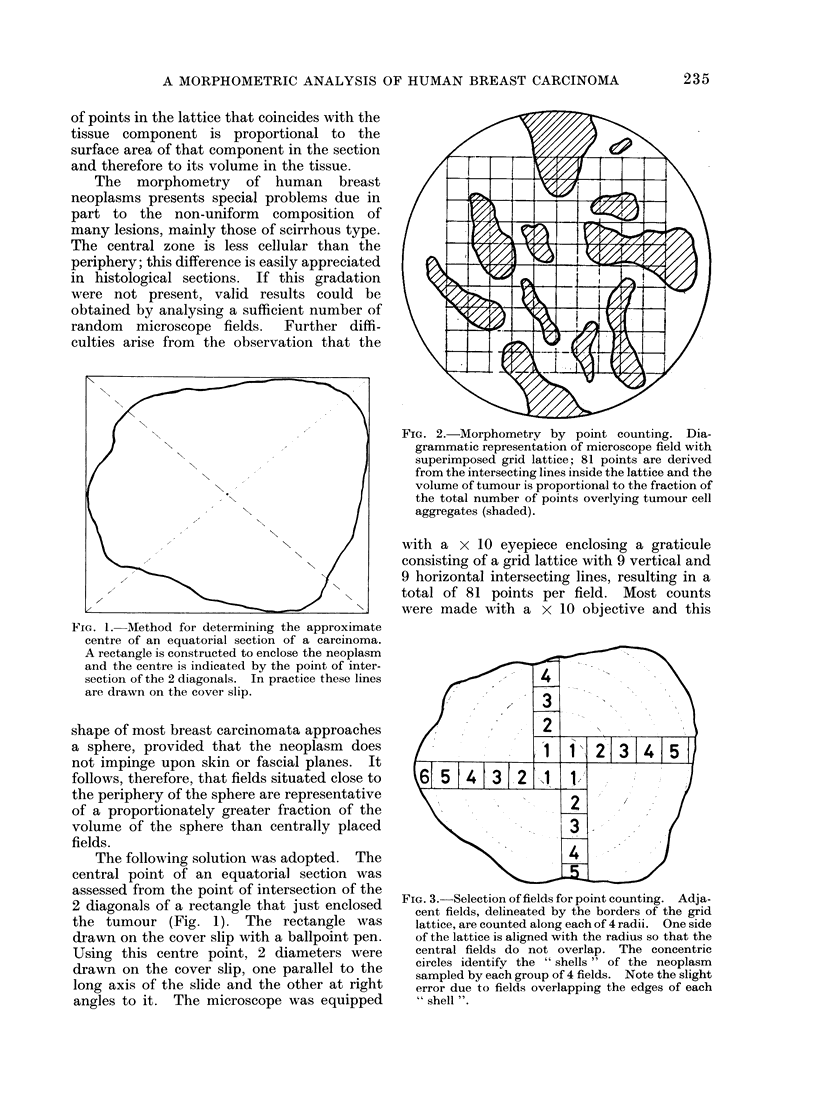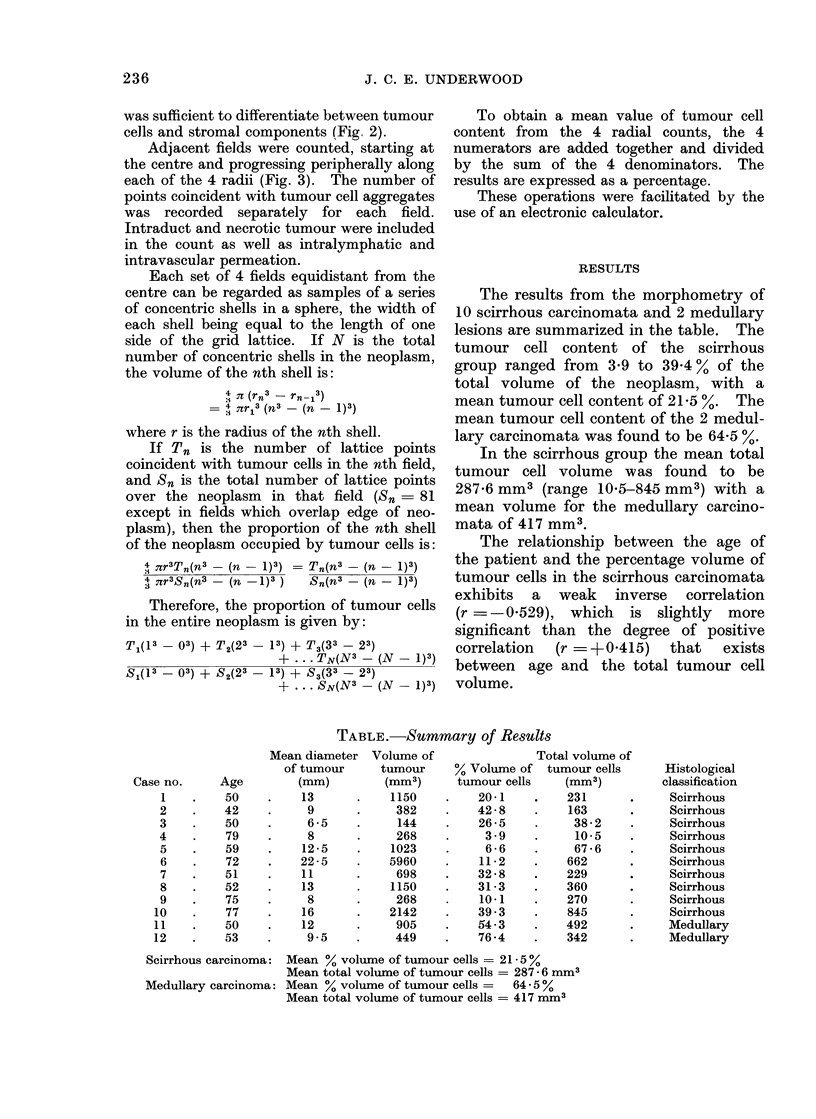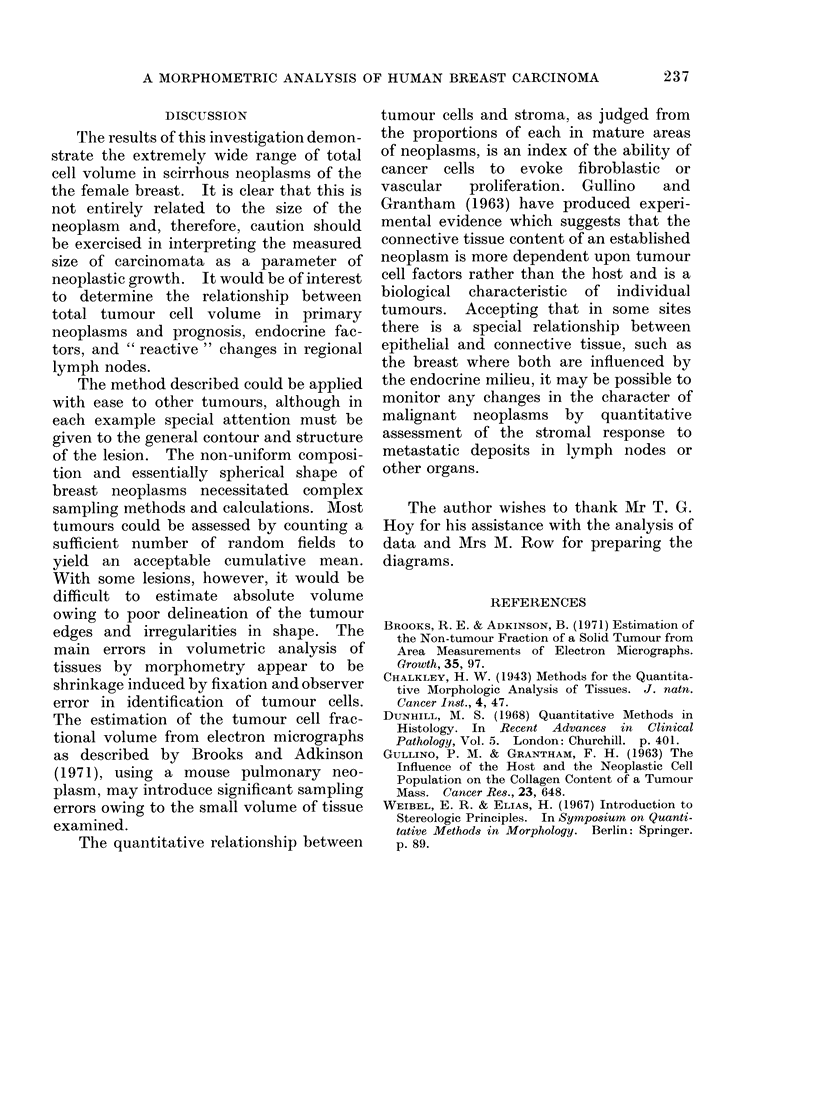# A Morphometric Analysis of Human Breast Carcinoma

**DOI:** 10.1038/bjc.1972.32

**Published:** 1972-06

**Authors:** J. C. E. Underwood

## Abstract

A method for determining the tumour cell content of breast neoplasms by morphometry is described.

The mean total tumour cell volume of 10 scirrhous carcinomata was 287·6 mm^3^, or 21·5% of the entire neoplasm. Two medullary lesions gave a mean tumour cell volume of 417 mm^3^, 64·5% of the total volume of the neoplasm.

A possible extension of this technique to monitor changes in the character of malignant tumours is discussed.


*Br. J. Cancer* (1972) **26**, 234

### A MORPHOMETRIC ANALYSIS OF HUMAN BREAST CARCINOMA

J. C. E. UNDERWOOD

*Department of Pathology, Sheffield University*

Received for publication December 1971

**Summary.—A method for determining the tumour cell content of breast neoplasms by morphometry is described.**

**The mean total tumour cell volume of 10 scirrhous carcinomata was 287·6 mm$^3$, or 21·5% of the entire neoplasm. Two medullary lesions gave a mean tumour cell volume of 417 mm$^3$, 64·5% of the total volume of the neoplasm.**

**A possible extension of this technique to monitor changes in the character of malignant tumours is discussed.**

THE principles and methods of morphometry, and the derivation of quantitative morphological data from tissues or tissue sections, have been well documented (Weibel and Elias, 1967; Dunhill, 1968) but the application of these techniques to the study of cancer has received little attention.

This paper gives an account of a method devised to determine the volumetric composition of human breast carcinoma. The volume of tumour cells within a series of breast neoplasms has been estimated and the relevance of this type of investigation to the study of malignant disease is discussed.

### MATERIALS AND METHODS

### *Breast tumours*

Ten examples of scirrhous carcinoma and 2 lesions classified as medullary carcinoma of the female breast were selected from routine surgical specimens at the Royal Hospital and Royal Infirmary, Sheffield. For reasons given below it was important to ensure that the block of tumour taken for histology was, as far as could be judged, an equatorial slice (*i.e.* it passed close to the centre of the tumour). The slice included the entire cut surface of the neoplasm in that plane. After fixation in 10% formol saline, paraffin sections were cut at 5–6 $\mu$m. With large tumours the equatorial slice was divided into 2 or more blocks for processing. Morphometry was carried out on sections stained by haematoxylin and eosin. Attempts to obtain clearer differentiation between tumour cells and stromal components by employing connective tissue stains resulted in a slight loss of the ability to distinguish tumour cells on cytological grounds. In addition, the rather immature connective tissue in some regions gave atypical staining reactions. The 12 breast carcinomata examined were selected only by adequacy of fixation.

In the text that follows, the word " neoplasm " is used only to describe the gross tumour, and this will avoid confusion with " tumour cells ", meaning a population of malignant cells.

### *Morphometry by point counting*

The Delesse principle, which forms the basis of volumetric analysis of tissues by morphometry, states that the volume of a discrete component (in this case tumour cells and cell aggregates) in a material (the neoplasm) can be estimated by measuring the area of a random section of the material that is occupied by that component. Of all the methods which have been devised for estimating the area of components in tissue sections, point counting is the most rapid and simple method that achieves a high degree of accuracy. Chalkley (1943) was the first to apply this procedure to histology. In practice, a regular point lattice is superimposed on the section, usually by mounting a graticule in the focal plane of a microscope eyepiece. The fraction of the total number of points in the lattice that coincides with the tissue component is proportional to the surface area of that component in the section and therefore to its volume in the tissue.

The morphometry of human breast neoplasms presents special problems due in part to the non-uniform composition of many lesions, mainly those of scirrhous type. The central zone is less cellular than the periphery; this difference is easily appreciated in histological sections. If this gradation were not present, valid results could be obtained by analysing a sufficient number of random microscope fields. Further difficulties arise from the observation that the shape of most breast carcinomata approaches a sphere, provided that the neoplasm does not impinge upon skin or fascial planes. It follows, therefore, that fields situated close to the periphery of the sphere are representative of a proportionately greater fraction of the volume of the sphere than centrally placed fields.

FIG. 1.—Method for determining the approximate centre of an equatorial section of a carcinoma. A rectangle is constructed to enclose the neoplasm and the centre is indicated by the point of intersection of the 2 diagonals. In practice these lines are drawn on the cover slip.

The following solution was adopted. The central point of an equatorial section was assessed from the point of intersection of the 2 diagonals of a rectangle that just enclosed the tumour (Fig. 1). The rectangle was drawn on the cover slip with a ballpoint pen. Using this centre point, 2 diameters were drawn on the cover slip, one parallel to the long axis of the slide and the other at right angles to it. The microscope was equipped with a × 10 eyepiece enclosing a graticule consisting of a grid lattice with 9 vertical and 9 horizontal intersecting lines, resulting in a total of 81 points per field. Most counts were made with a × 10 objective and this

FIG. 2.—Morphometry by point counting. Diagrammatic representation of microscope field with superimposed grid lattice; 81 points are derived from the intersecting lines inside the lattice and the volume of tumour is proportional to the fraction of the total number of points overlying tumour cell aggregates (shaded).

FIG. 3.—Selection of fields for point counting. Adjacent fields, delineated by the borders of the grid lattice, are counted along each of 4 radii. One side of the lattice is aligned with the radius so that the central fields do not overlap. The concentric circles identify the " shells " of the neoplasm sampled by each group of 4 fields. Note the slight error due to fields overlapping the edges of each " shell ".

was sufficient to differentiate between tumour cells and stromal components (Fig. 2).

Adjacent fields were counted, starting at the centre and progressing peripherally along each of the 4 radii (Fig. 3). The number of points coincident with tumour cell aggregates was recorded separately for each field. Intraduct and necrotic tumour were included in the count as well as intralymphatic and intravascular permeation.

Each set of 4 fields equidistant from the centre can be regarded as samples of a series of concentric shells in a sphere, the width of each shell being equal to the length of one side of the grid lattice. If $N$ is the total number of concentric shells in the neoplasm, the volume of the $n$th shell is:

$$\begin{aligned} &\tfrac{4}{3}\,\pi\,(r_n{}^3 - r_{n-1}{}^3) \\ &= \tfrac{4}{3}\,\pi r_1{}^3\,(n^3 - (n-1)^3) \end{aligned}$$

where $r$ is the radius of the $n$th shell.

If $T_n$ is the number of lattice points coincident with tumour cells in the $n$th field, and $S_n$ is the total number of lattice points over the neoplasm in that field ($S_n = 81$ except in fields which overlap edge of neoplasm), then the proportion of the $n$th shell of the neoplasm occupied by tumour cells is:

$$\frac{\tfrac{4}{3}\,\pi r^3 T_n(n^3 - (n-1)^3)}{\tfrac{4}{3}\,\pi r^3 S_n(n^3 - (n-1)^3)} = \frac{T_n(n^3 - (n-1)^3)}{S_n(n^3 - (n-1)^3)}$$

Therefore, the proportion of tumour cells in the entire neoplasm is given by:

$$\frac{T_1(1^3 - 0^3) + T_2(2^3 - 1^3) + T_3(3^3 - 2^3) + \ldots T_N(N^3 - (N-1)^3)}{S_1(1^3 - 0^3) + S_2(2^3 - 1^3) + S_3(3^3 - 2^3) + \ldots S_N(N^3 - (N-1)^3)}$$

To obtain a mean value of tumour cell content from the 4 radial counts, the 4 numerators are added together and divided by the sum of the 4 denominators. The results are expressed as a percentage.

These operations were facilitated by the use of an electronic calculator.

### RESULTS

The results from the morphometry of 10 scirrhous carcinomata and 2 medullary lesions are summarized in the table. The tumour cell content of the scirrhous group ranged from 3·9 to 39·4% of the total volume of the neoplasm, with a mean tumour cell content of 21·5%. The mean tumour cell content of the 2 medullary carcinomata was found to be 64·5%.

In the scirrhous group the mean total tumour cell volume was found to be 287·6 mm³ (range 10·5–845 mm³) with a mean volume for the medullary carcinomata of 417 mm³.

The relationship between the age of the patient and the percentage volume of tumour cells in the scirrhous carcinomata exhibits a weak inverse correlation ($r = -0{\cdot}529$), which is slightly more significant than the degree of positive correlation ($r = +0{\cdot}415$) that exists between age and the total tumour cell volume.

TABLE.—*Summary of Results*

| Case no. | Age | Mean diameter of tumour (mm) | Volume of tumour (mm³) | % Volume of tumour cells | Total volume of tumour cells (mm³) | Histological classification |
|---|---|---|---|---|---|---|
| 1 | 50 | 13 | 1150 | 20·1 | 231 | Scirrhous |
| 2 | 42 | 9 | 382 | 42·8 | 163 | Scirrhous |
| 3 | 50 | 6·5 | 144 | 26·5 | 38·2 | Scirrhous |
| 4 | 79 | 8 | 268 | 3·9 | 10·5 | Scirrhous |
| 5 | 59 | 12·5 | 1023 | 6·6 | 67·6 | Scirrhous |
| 6 | 72 | 22·5 | 5960 | 11·2 | 662 | Scirrhous |
| 7 | 51 | 11 | 698 | 32·8 | 229 | Scirrhous |
| 8 | 52 | 13 | 1150 | 31·3 | 360 | Scirrhous |
| 9 | 75 | 8 | 268 | 10·1 | 270 | Scirrhous |
| 10 | 77 | 16 | 2142 | 39·3 | 845 | Scirrhous |
| 11 | 50 | 12 | 905 | 54·3 | 492 | Medullary |
| 12 | 53 | 9·5 | 449 | 76·4 | 342 | Medullary |

Scirrhous carcinoma: Mean % volume of tumour cells = 21·5%
Mean total volume of tumour cells = 287·6 mm³
Medullary carcinoma: Mean % volume of tumour cells = 64·5%
Mean total volume of tumour cells = 417 mm³

### DISCUSSION

The results of this investigation demonstrate the extremely wide range of total cell volume in scirrhous neoplasms of the the female breast. It is clear that this is not entirely related to the size of the neoplasm and, therefore, caution should be exercised in interpreting the measured size of carcinomata as a parameter of neoplastic growth. It would be of interest to determine the relationship between total tumour cell volume in primary neoplasms and prognosis, endocrine factors, and " reactive " changes in regional lymph nodes.

The method described could be applied with ease to other tumours, although in each example special attention must be given to the general contour and structure of the lesion. The non-uniform composition and essentially spherical shape of breast neoplasms necessitated complex sampling methods and calculations. Most tumours could be assessed by counting a sufficient number of random fields to yield an acceptable cumulative mean. With some lesions, however, it would be difficult to estimate absolute volume owing to poor delineation of the tumour edges and irregularities in shape. The main errors in volumetric analysis of tissues by morphometry appear to be shrinkage induced by fixation and observer error in identification of tumour cells. The estimation of the tumour cell fractional volume from electron micrographs as described by Brooks and Adkinson (1971), using a mouse pulmonary neoplasm, may introduce significant sampling errors owing to the small volume of tissue examined.

The quantitative relationship between tumour cells and stroma, as judged from the proportions of each in mature areas of neoplasms, is an index of the ability of cancer cells to evoke fibroblastic or vascular proliferation. Gullino and Grantham (1963) have produced experimental evidence which suggests that the connective tissue content of an established neoplasm is more dependent upon tumour cell factors rather than the host and is a biological characteristic of individual tumours. Accepting that in some sites there is a special relationship between epithelial and connective tissue, such as the breast where both are influenced by the endocrine milieu, it may be possible to monitor any changes in the character of malignant neoplasms by quantitative assessment of the stromal response to metastatic deposits in lymph nodes or other organs.

The author wishes to thank Mr T. G. Hoy for his assistance with the analysis of data and Mrs M. Row for preparing the diagrams.

### REFERENCES

Brooks, R. E. & Adkinson, B. (1971) Estimation of the Non-tumour Fraction of a Solid Tumour from Area Measurements of Electron Micrographs. *Growth*, **35**, 97.

Chalkley, H. W. (1943) Methods for the Quantitative Morphologic Analysis of Tissues. *J. natn. Cancer Inst.*, **4**, 47.

Dunhill, M. S. (1968) Quantitative Methods in Histology. In *Recent Advances in Clinical Pathology*, Vol. 5. London: Churchill. p. 401.

Gullino, P. M. & Grantham, F. H. (1963) The Influence of the Host and the Neoplastic Cell Population on the Collagen Content of a Tumour Mass. *Cancer Res.*, **23**, 648.

Weibel, E. R. & Elias, H. (1967) Introduction to Stereologic Principles. In *Symposium on Quantitative Methods in Morphology*. Berlin: Springer. p. 89.